# Notes on the genus *Harmonicon* F.O.P.-Cambridge, 1896 (Araneae, Dipluridae) with description of a new species from French Guyana

**DOI:** 10.3897/zookeys.112.1205

**Published:** 2011-06-24

**Authors:** Bastian Drolshagen, Christian M. Bäckstam

**Affiliations:** 1 Institute of Environmental Sciences, University of Koblenz-Landau, Fortstrasse 7, 76829 Landau, Germany; 2 Olshammarsgatan 36, S-12475 Bandhagen, Sweden

**Keywords:** spider taxonomy, diplurinae, new species, typus rediscovery

## Abstract

Information on the genus *Harmonicon* F.O.P.-Cambridge, 1896, a key to the species and a new diagnosis differing from the one in Maréchal and Marty (1998) are provided. A new species is described: *Harmonicon oiapoqueae* differing from other species of the genus by the morphology of the posterior sternal sigilla, the more recurved, inverted U–shaped fovea, the amount and arrangement of maxillary cuspules, a single row of teeth on the claws of the palpal tarsus, longer and more slender legs III and IV in females, longer embolus, thinner bulb, and longer, more slender legs in males. The status of the putative junior synonyms of *Harmonicon*, *Pseudohermachura *Mello-Leitão, 1927 and *Prosharmonicon* Mello-Leitão, as well as the two species formerly assigned to *Harmonicon*, *Harmonicon nigridorsi *Mello-Leitão, 1924 and *Harmonicon riveti *Simon, 1903, is discussed.

## Introduction

The genus *Harmonicon *F.O.P.-Cambridge, 1896 was established on the basis of a single juvenile specimen with the type species *Harmonicon rufescens* F.O.P.-Cambridge, 1896 from Santarem, Brazil. Raven (1985) considered *Harmonicon* a junior synonym of *Diplura *C.L. Koch, 1850 because both genera possess a lyra consisting of few modified bristles on the prolateral side of the maxillae. Maréchal and Marty (1998) rejected this synonymy on the basis of the shape of the lyra bristles that differ between the two genera and a different leg formula, and they described an additional species: *Harmonicon audeae *Maréchal & Marty, 1998.

## Material and methods

Material and methods follow Drolshagen and Bäckstam (2009). Abbreviations and measurement of male palpal organ follow Coyle (1995): PL = length of male palpal organ, BD = bulbus width. Measurements and leg proportion (diameter of femur/length of leg × 100) follow Maréchal and Marty (1998). The term megaspine is used according to Raven (1984). Additional abbreviations: imm = immature, Co = coxa, Fe = femur, Pa = patella, Ti = tibia, Mt = metatarsus, Ta = tarsus, STC = superior tarsal claw, ITC = inferior tarsal claw, PMS = posterior median spinnerets, PLS = posterior lateral spinnerets.

### Acronyms of institutes, museums and collections:

IBSP:Instituto Butantã, São Paulo; SMNK:Staatliches Museum für Naturkunde Karlsruhe; MNHN:Muséum National d’Histoire Naturelle, Paris; MPSP:Universidade de São Paulo, Museu Paulista; NHM:The Natural History Museum, London (formerly British Museum, Natural History); NHMW:Naturhistorisches Museum, Wien; SMF:Forschungsinstitut und Naturmuseum Senckenberg, Frankfurt am Main; PCD = Drolshagen private collection.

Examined (type-) material: *Diplura nigra* (F. O. P.-Cambridge, 1896), female holotype, NHM (BMNH1896.12.13.49), Santarem, lower Amazonas, Brazil. *Diplura sanguinea* (F. O. P.-Cambridge, 1896), female holotype, NHM (BMNH1896.12.13.41), Santarem, lower Amazonas, Brazil. *Harmonicon rufescens* F. O. P.-Cambridge, 1896, imm male holotype, NHM (no collection number), Santarem, lower Amazonas, Brazil. *Harmonicon oiapoqueae* sp. n. male holotype and paratype female, SMNK, Saint Georges, French Guiana.*Harmonicon oiapoqueae* sp. n. juvenile, PCD, Saint Georges, French Guiana.*Trechona rogenhoferi* (Ausserer, 1871), female holotype, NHMW (N.I.: 62), Brazil. *Trechona rufa* (Ausserer, 1871), female SMF (Nr. 38604), Miracatu, São Paulo, Brazil. *Trechona zebrata* (Walkenaer, 1835) (currently in synonymy of *Trechona venosa *(Latreille, 1832)), female holotype, NHM (no collection number), Brazil.

## Taxonomy

### Harmonicon

F.O.P.-Cambridge, 1896

http://species-id.net/wiki/Harmonicon

Harmonicon F.O.P.-Cambridge 1896: 755; Maréchal and Marty 1998: 500.

#### Diagnosis:

*Harmonicon *is one of the genera of the subfamily Diplurinae with a lyra prolaterally on the maxillae. It differs from *Diplura* by the shape of the lyra bristles (claviform in *Diplura* rather than hookshaped in *Harmonicon*), a more dense scopula on Ta I and II, the presence of a dense scopula in more than the apical third of pedipalpal tarsus, the presence of a scopula in the apical third of most leg metatarsi, and by the leg tarsi being pseudosegmented instead of showing only a few cracks. *Harmonicon* can be distinguished from *Trechona* by a less dense scopula on Ta III and IV, and by fewer lyra bristles arranged in a single row.

#### Remarks:

 Cambridge (1896) and Maréchal and Marty (1998) regarded the presence of five lyra bristles a key feature for the genus. Research on the development on the new species described here showed that younger specimens have fewer lyra bristles than fully grown ones. Therefore the number of lyra bristles should no longer be considered diagnostic for *Harmonicon*. Maréchal and Marty (1998) stated that the leg formula of 1423 (rather than 4123 as in *Diplura* and *Trechona* C.L. Koch, 1850), is also a key feature of the genus *Harmonicon*, but show a leg formula of 4123 for the female holotype of *Harmonicon audeae*. Cambridge (1896) was incorrect in stating that the holotype of *Harmonicon rufescens* is a female; it is in fact a juvenile male. Maréchal and Marty (1998) did not comment on the putative synonyms of *Harmonicon*, *Pseudohermachura* Mello-Leitão, 1927 and *Prosharmonicon* Mello-Leitão, 1938, as well as those species formerly assigned to *Harmonicon*: *Harmonicon nigridorsi* Mello-Leitão, 1924 and *Harmonicon riveti* Simon, 1903. Mello-Leitão (1927) described the monotypical genus *Pseudohermachura* from a single female specimen of the type species *Pseudohermachura catharinensis* Mello-Leitão, 1927 (holotype deposited in MPSP) and did not mention the presence of a lyra at all. Bücherl (1962) redescribed *Pseudohermachura catharinensis* and mentioned a lyra consisting of 7–10 claviform bristles. Mello-Leitão (1938) described the monotypical genus *Prosharmonicon* from a single female specimen of the type species *Prosharmonicon maculatum* Mello-Leitão, 1938 (holotype deposited in IBSP and destroyed in the fire of 2010) and explicitly mentioned claviform lyra bristles. We therefore consider *Pseudohermachura* and *Prosharmonicon* junior synonyms of *Diplura* and reject the synonymies with *Harmonicon* established by Bücherl (1962). Simon (1903) described *Harmonicon riveti* from a single male specimen, of which the palpal organ, distal part of Ti I and basal part of Mt I were later illustrated in Berland (1913): pl. 7, fig. 5–6. The illustrations show a palpal organ with a strongly curved apex of the embolus and a highly elevated tubercle laterally in the basal third of Mt I. The morphology of the palpal organ and the tubercle in the basal third of Mt I in (known) males is different in those species currently assigned to *Harmonicon*. Mello-Leitão (1924) described *Harmonicon nigridorsi* from a single female specimen, he explicitly mentioned claviform lyra bristles, which is also supported by the illustration in Mello-Leitão (1926): fig. 4. We therefore support the transfer of those two species to *Diplura* by Raven (1985).

#### Key to the species of *Harmonicon*

**Table:** 

1	Female or juvenile	2
–	Male	4
2	Tarsal claw of pedipalps with one row of teeth	3
–	Tarsal claw of pedipalps with a double row of teeth	*Harmonicon audeae*
3	Fovea slightly recurved, not inverted U–shaped; approximately 30–40 maxillary cuspules; posterior pair of sternal sigilla circular	*Harmonicon rufescens*
–	Fovea strongly recurved, inverted U–shaped; approximately 40–50 maxillary cuspules; posterior pair of sternal sigilla oval	*Harmonicon oiapoqueae* sp. n.
4	Palpal organ long, bulbus narrow [PL(100)/BD = 251]; tubercle in basal third of metatarsus I absent	*Harmonicon audeae*
–	Palpal organ short, bulbus wide [PL(100)/BD = 147]; tubercle in basal third of metatarsus I present	*Harmonicon oiapoqueae* sp. n.

### Harmonicon oiapoqueae sp. n.

urn:lsid:zoobank.org:act:AAE8BAB3-42C4-4A88-B2F6-ADCA489482B4

http://species-id.net/wiki/Harmonicon_oiapoqueae

#### Type material:

Male holotype and 1 female paratype (SMNK) from Saint Georges, French Guiana, 3°56'56.12"N, 51°47'39.90"W (leg. T. Vinmann).

#### Other material examined:

PCD–33–306–03 1 imm of *Harmonicon oiapoqueae*, same data as for holotype and paratype.

#### Etymology:

The specific epithet, a feminine genitive singular, refers to the Oiapoque river, which is close to the type locality.

#### Diagnosis:

*Harmonicon oiapoqueae* sp. n. can be distinguished from the other species of the genus by the posterior pair of sternal sigilla being oval instead of circular. It furthermore differs from *Harmonicon audeae* by only one row of teeth on the tarsal claws of the pedipalp and from *Harmonicon rufescens* by a more stronly recurved, inverted U–shaped fovea and position and arrangement of cuspules on the basal inner corner of the maxillae. *Harmonicon oiapoqueae* sp. n. differs from *Harmonicon audeae* and *Harmonicon rufescens* by more slender legs III and IV in females and juveniles and legs I-IV in males. Furthermore, males can be distinguished from those of *Harmonicon audeae* by a shorter embolus and a wider bulbus [PL(100)/BD = 147] and the presence of a tubercle in the basal third of the lateral metatarsus I.

#### Description:

##### Male holotype:

**Figures 1–7.**
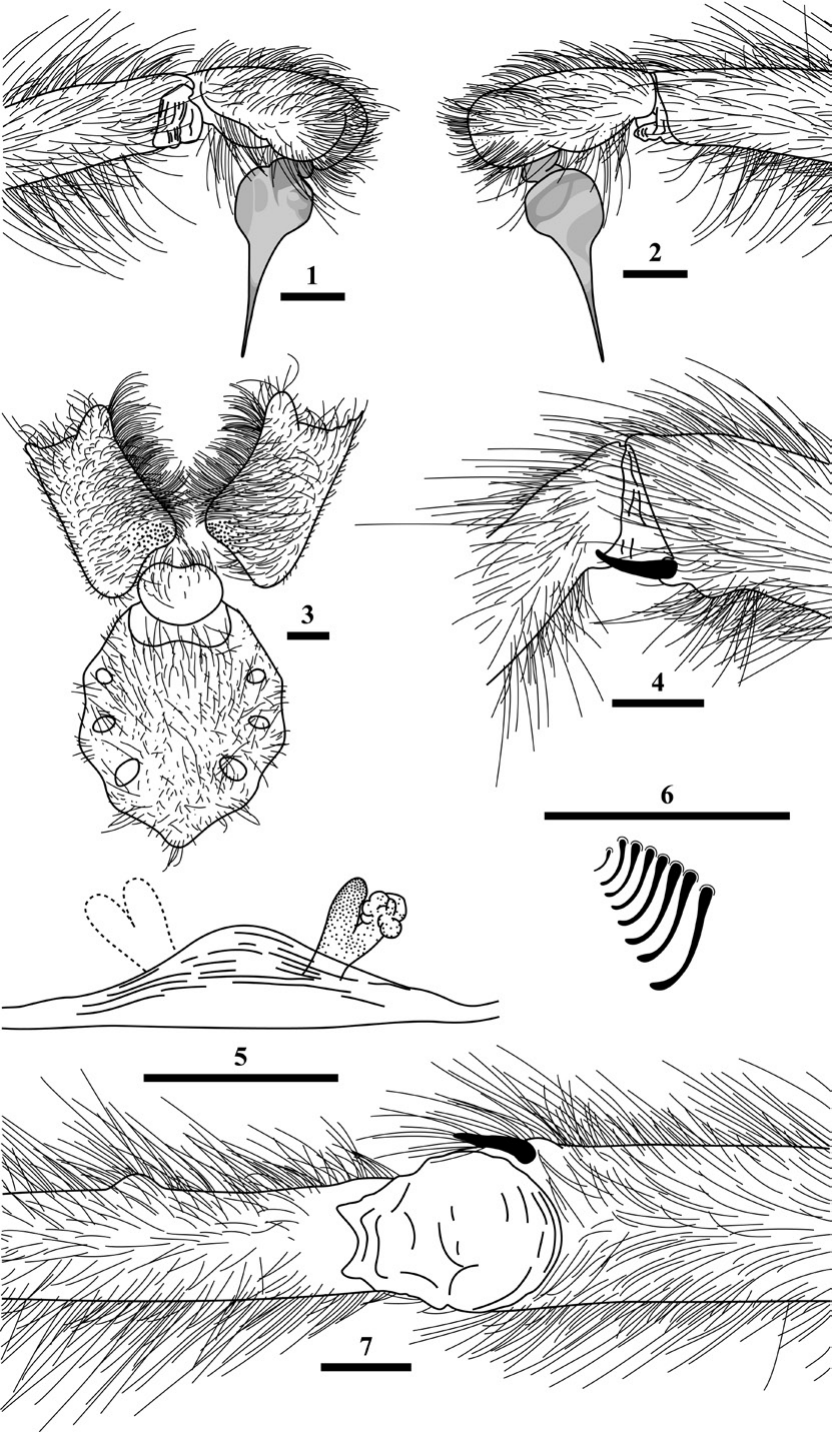
*Harmonicon oiapoqueae* sp. n. **1, 2, 4, 7** male holotype; **3, 5, 6** female paratype. **1** right palp, retrolateral view **2** right palp, prolateral view **3** sternum, labium and maxillae, ventral view **4** distal part of Ti and basal part of Mt I, retrolateral view **5** vulva (with left receptaculum seminis lost), dorsal view **6** lyra **7** distal part of Ti and basal part of Mt I, ventral view. Scale bars = 1 mm each.

Colour in alcohol: carapace, legs and pedipalps mahagony brown, chelicerae red, opisthosoma grey. Carapace (length: 11.67; width: 10.38) covered with soft grey setae and longer, black setae in posterior thoracic area; margin with long, black setae and soft, silver setae; clypeus present, narrow; fovea slitlike, recurved, inverted U–shaped; striae marked. Chelicerae with two retrolateral bands of plumose setae and one dorsal band, broadening to full width of chelicerae distally; ventrally with one row of 9 teeth (1–1–1–1–5) on promargin; cheliceral furrow with a field of small basomesal teeth; retroventral base with isolated bristles. Maxillae with prolateral lyra consisting of 7 hookshaped bristles (as in the female paratype - viz. Fig. 6); ventrally with few cuspules on basal inner corner, number and arrangement different in both sides. Labium trapezoidal, without cuspules; labiosternal suture short and divided. Sternum with 3 pairs of sigilla: anterior pair at height of Co I, circular, medial pair at Co II, circular, posterior pair between Co III and IV, oval, largest; anterior and medial pairs almost equal in size. Legs long and slender (measurements and proportions in table 1), with all tarsi pseudosegmented (Ta IV missing). All present tarsi with dense and entire scopula; metatarsi also scopulated in apical third, but less dense. STC at Ta I and II truncated (maybe worn off), not curved; normal at Ta III, curved and long; all with few teeth; ITC short, without teeth. Ti I retroventrally at apex with megaspine (Fig. 4, 7); Mt I with a low, domed tubercle retroventrally in basal third (Fig. 7). Number and position of spines on legs different on both sides. Opisthosoma (length: 12.34; width: 7.15) with two pairs of spinnerets: PMS small, consisting of one segment (length: 3.96), widely separated from each other. PLS elongated, consisting of three segments: basal (length: 5.62), medial (length: 6.18), apical (length: 10.68) longest. Palpal bulb pyriform with relatively long, almost straight embolus (Figs 1, 2).

##### Female paratype:

Colour in alcohol: resembles male holotype, but legs darker. Carapace (length: 13.26; width: 12.54) and opisthosoma (length: 17.37; width: 7.58) larger than that of holotype. Different from male holotype by the presence of one more labial cuspule and several more cuspules on basal inner corner of maxillae (Fig. 3). Chelicerae with more teeth on promargin (1–1–1–1–1–1–1–1–3). Lyra as in Fig. 6. Legs not as long and slender (measurements and proportions in table 1 in parenthesis). Pedipalpal tarsus with slight scopula, divided by two parallel rows of spiniform setae in apical third, becoming more irregular beyond apical third. Slight scopula on Ta I and II; divided by two parallel rows of spiniform setae. Mt I and II with scopula less dense only covering apical third, divided by two parallel rows of spiniform setae, becoming more irregular basally. Ta III like Ta I and II; Mt III without scopula, only with hairlike setae. Ta IV with only thin scopula and more setae, Mt IV like Mt III. Leg spination as in male holotype. Opisthosoma resembles male holotype, but with PMS (length: 3.27) more widely (by length of segment) seperated and basal segment of PLS (length: 5.12), medial (length: 4.44), apical (length: 8.55) slightly shorter. Vulva as in Fig. 5.

#### Remarks:

Although the number of lyra bristles varies during the development of this species, the paratype of *Harmonicon oiapoqueae* sp.nov. has more such bristles than the female holotype of *Harmonicon audeae*; both specimens are of almost the same size.

#### Ecology:

According to Thomas Vinmann (pers. comm.), who collected the specimens examined, females build large sheetwebs of *ca*. 2 m2 which are attached to branches of trees and bushes. The sheetweb runs into a funnel which leads to a ca. 20 cm deep tube–shaped retreat. The burrow leads about 5 cm vertically into the ground and continues for *ca*. 10 cm at an angle of about 45°. At dusk the spiders come to the entrance of the funnel and wait for prey.

**Table 1.** Measurements of male holotype and female paratype (in parenthesis) of *Harmonicon oiapoqueae* sp. n. legs, pedipalps and proportions

	Leg I	Leg II	Leg III	Leg IV	Pedipalp
Fe	17.96 (13.34)	15.05 (12.54)	13.34 (11.18)	15.77 (13.47)	8.05 (7.95)
Pa	6.75 (6.58)	5.52 (6.06)	3.70 (5.19)	5.05 (6.05)	3.31 (4.13)
Ti	15.38 (10.50)	12.79 (10.08)	13.54 (8.38)	13.77 (10.89)	6.28 (6.23)
Mt	17.65 (10.27)	15.59 (10.05)	15.11 10.48)	17.69 (14.27)	-
Ta	10.63 (7.03)	8.91 (6.89)	8.19 (6.44)	- (7.24)	3.12 (6.58)
Total	68.37 (47.72)	57.86 (45.62)	53.88 (41.67)	52.28 (51.92)	20.76 (24.89)
Diameter of Fe	2.61 (2.97)	2.51 (2.91)	2.38 (2.58)	2.47 (2.83)	-
Proportion	3.82 (6.22)	4.34 (6.38)	4.42 (6.84)	- (5.45)	-

## Supplementary Material

XML Treatment for Harmonicon

XML Treatment for Harmonicon oiapoqueae
